# Supplementary material for: Comparative multiomics analysis of cell physiological state after culture in a basket bioreactor
Source: Sci Rep. 2022 Nov 23;12:20161. doi: 10.1038/s41598-022-24687-4 (PMC9686226; doi:10.1038/s41598-022-24687-4)
Supplement: Supplementary file 1 — Supplementary Information 1. [file 41598_2022_24687_MOESM1_ESM.zip › raw data/Transcriptomic raw data/4.Quant/2.Correlation/correlation.pdf]

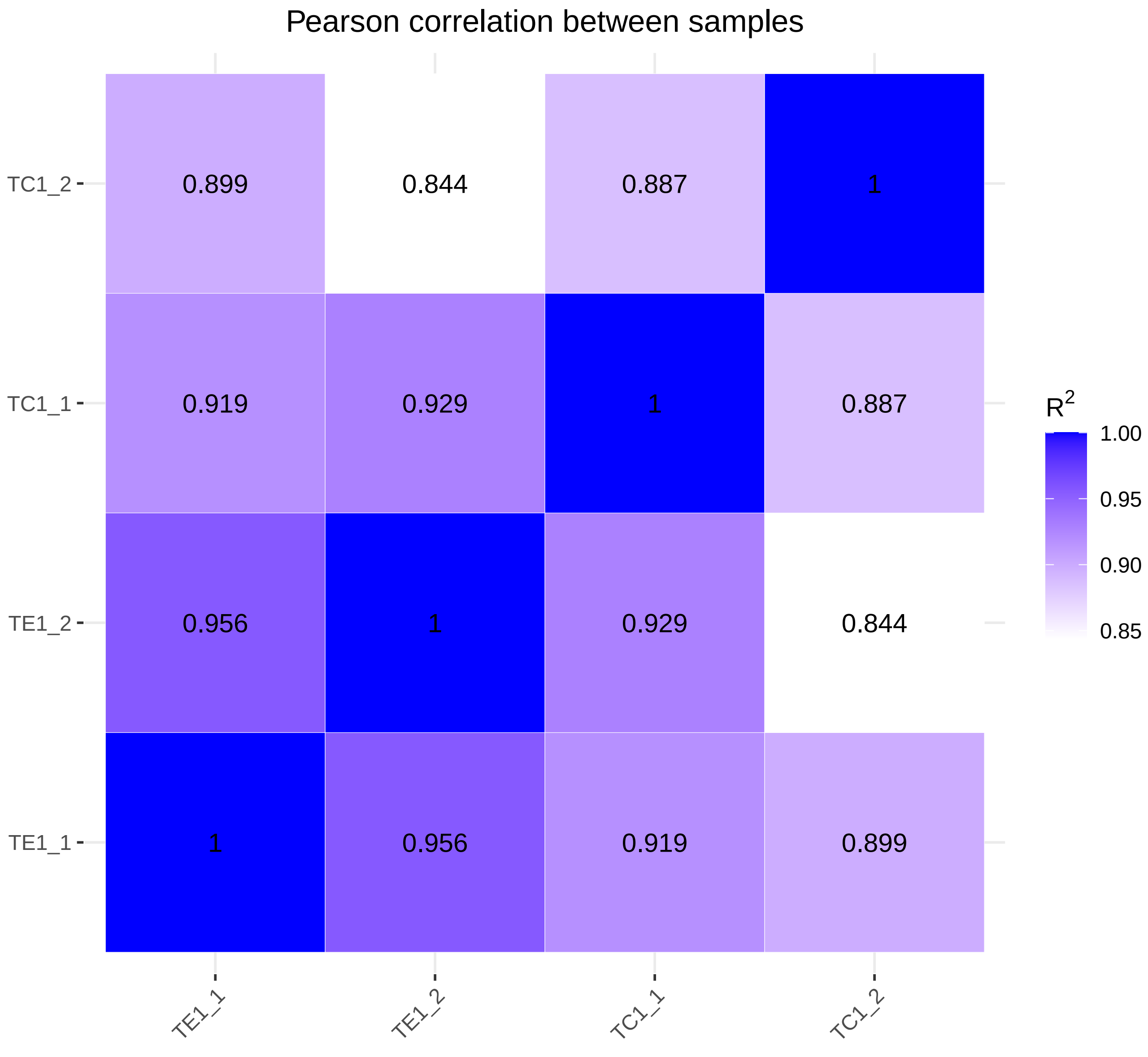
Pearson correlation between samples
TC1_2
TC1_1
TE1_2
TE1_1
0.899
0.844
0.887
1
0.919
0.929
1
0.887
0.956
1
0.929
0.844
1
0.956
0.919
0.899
TE1_1
TE1_2
TC1_1
TC1_2
R2
1.00
0.95
0.90
0.85
